# Integrator is a key component of human telomerase RNA biogenesis

**DOI:** 10.1038/s41598-018-38297-6

**Published:** 2019-02-08

**Authors:** M. P. Rubtsova, D. P. Vasilkova, M. A. Moshareva, A. N. Malyavko, M. B. Meerson, T. S. Zatsepin, Y. V. Naraykina, A. V. Beletsky, N. V. Ravin, O. A. Dontsova

**Affiliations:** 1 Center of Life Sciences, Skolkovo Institute of Science and Technology, Skolkovo, Moscow 143026 Russia; 2 Lomonosov Moscow State University, Department of Chemistry, Faculty of Bioengineering and Bioinformatics and A.N. Belozersky Institute of Physico-Chemical Biology, Moscow, 119992 Russia; 3 Institute of Bioengineering, Research Center of Biotechnology of the Russian Academy of Sciences, Moscow, 119071 Russia; 4 Shemyakin-Ovchinnikov Institute of Bioorganic Chemistry of the Russian Academy of Sciences, Moscow, 117997 Russia

## Abstract

Telomeres are special DNA-protein structures that are located at the ends of linear eukaryotic chromosomes. The telomere length determines the proliferation potential of cells. Telomerase is a key component of the telomere length maintenance system. While telomerase is inactive in the majority of somatic cells, its activity determines the clonogenic potential of stem cells as a resource for tissue and organism regeneration. Reactivation of telomerase occurs during the process of immortalization in the majority of cancer cells. Telomerase is a ribonucleoprotein that contains telomerase reverse transcriptase and telomerase RNA components. The RNA processing mechanism of telomerase involves exosome trimming or degradation of the primary precursor. Recent data provide evidence that the competition between the processing and decay of telomerase RNA may regulate the amount of RNA at the physiological level. We show that termination of human telomerase RNA transcription is dependent on its promoter, which engages with the multisubunit complex Integrator to interact with RNA polymerase II and terminate transcription of the human telomerase RNA gene followed by further processing.

## Introduction

Telomerase promotes the proliferative potential of cells due to the lengthening of telomeres, which are special DNA regions located at the ends of linear eukaryotic chromosomes^1^. Telomerase activity is inactivated during cellular differentiation, and the majority of somatic cells of the human organism do not possess active telomerase^2^. Telomerase reverse transcriptase synthesizes telomeres using the telomerase RNA template region^3,4^. Inactivation of telomerase during cellular differentiation occurs due to silencing of the expression of the hTERT gene^2^. However, hTR gene expression is not shut off in the majority of somatic cells^5^, suggesting an additional functional role of hTR independent of hTERT^6–8^. Numerous proteins participate in telomerase biogenesis, including telomerase RNA processing, trafficking through cellular compartments, and the association of hTERT with hTR and telomeres. Defects in telomerase components and ancillary proteins cause diseases that involve the phenomenon of shortened telomeres, such as dyskeratosis congenita, aplastic anemia, idiopathic pulmonary fibrosis and bone marrow failure^9,10^.

Vertebrate telomerase RNAs contain a 3′-end domain common with H/ACA-RNAs that guide the site-specific pseudouridinylation of target RNAs^11^. However, the target for hTR is still unknown. The expression of hTR is driven by RNA polymerase II, but correct processing of hTR depends on promoter regulation of its transcription^9,10^. The hTR native promoter as well as the promoter of U3 snRNA facilitates the correct processing of hTR, while CMV promoter driven expression leads to the accumulation of unprocessed product^12^. The processing of hTR occurs in a transcriptionally dependent manner due to attraction of the exosome by the CBCN complex (cap-binding complex (CBC) with NEXT (CBCN)) to the primary transcript^13^. Fast hTR degradation performed by exosome trimming of hTR competes with processing events facilitated by the PARN1 exonuclease, which is recruited to the transcript by the CBCA complex (complex of CBC with ARS2 protein), as was determined recently^13^. PARN1 correctly processes hTR oligoadenylated by the TRAMP complex^13,14^. It was shown that CBCA is involved in the processing of the hTR primary transcript^13,14^ through the regulation of the recruitment of the exosome/TRAMP complexes^13^. However, the events that result in the appearance of the primary transcript need to be clarified.

RNA polymerase II-mediated transcription of particular RNA is regulated by the multisubunit complexes Mediator and Integrator. Mediator is responsible for mRNA transcription^15–17^, and Integrator is responsible for noncoding RNAs (snRNAs) and some specialized forms of mRNA transcription (histone mRNAs for example)^18–20^. Integrator is considered to be a functional analogue of Sen1 in yeast^20^, which is known to be involved in the transcription termination of sn- and snoRNAs genes. Therefore, Integrator is likely involved in the regulation of the transcription of hTR since hTR has features of snoRNA because of its H/ACA domain^11^. To test this hypothesis, we used a bicistronic reporter system with different promoters that control the transcription as well as the knockdown of particular Integrator subunits. Our results demonstrate that Integrator is indeed a key regulator of the transcription termination of hTR.

## Results

### The native promoter directs human telomerase RNA transcription termination to the proper position

**Figure 1:**
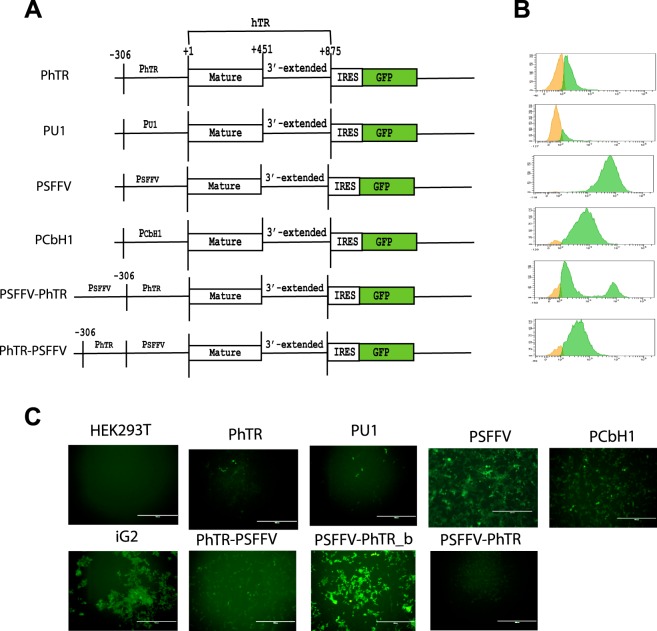
Transcription termination of human telomerase RNA depends on the promoter type. (**A**) Schematic representation of the hTR bicistronic reporters. (**B**) Expression of GFP revealed by flow cytometry analysis of the reporter cell lines. (**C**) Expression of GFP revealed by immunofluorescence microscopy of the reporter cell lines.

To investigate human telomerase RNA processing and transcription termination, we developed a reporter system based on a bicistronic construct. This construct contained the hTR genomic region that corresponds to the mature form of hTR flanked by 425 base pairs of downstream nucleotides. The IRES element and GFP-coding region were placed after the first cistron (Fig. 1A). GFP translation could occur only in the case of bicistronic mRNA synthesis. To analyze the influence of the promoter on hTR transcription, we used various constructs, in which hTR expression was regulated by different promoters (Fig. 1A), such as the SFFV and CbH1 promoters regulated by the Mediator complex^17^ and the U1 promoter known to be regulated by the Integrator complex^18^. The PhTR construct contained only the hTR promoter (300 nucleotides upstream of hTR), the PSFFV – SFFV promoter, the PU1 – U1snRNA promoter, the CbH1 – chicken b-actin gene promoter, and the PSFFV-hTR and PhTR-SFFV-coupled SFFV-hTR- or hTR-SFFV-promoters (Fig. 1A). These constructs were incorporated into the cellular genome of HEK293T cells through viral transduction. The cells containing the construct driven by the SFFV and CbH1 promoters demonstrated a bright GFP signal (Fig. 1B,C), whereas those with the construct under the hTR, U1 or coupled SFFV-hTR and hTR-SFFV promoters provided only a slight shift in fluorescent signal in comparison to wild-type HEK293T cells (Fig. 1B,C). Interestingly, the cells that expressed the bicistronic construct under the control of the coupled SFFV-hTR promoter were divided for two separate fluorescent populations during long-term cultivation (50 population doublings): original (PSFFV-hTR) and more intensive (PSFFV-hTR-b). The cells with a more intensive fluorescent signal were accumulated during passaging (Fig. 1B,C).

The variations in GFP fluorescence might have been caused by different efficiencies of viral cassette integration in the genomes of the infected cells. We performed the analysis of the copy number of the viral cassette integrated in the genome for each obtained cell line by qPCR analysis of purified genomic DNA using the primers specific to the GFP-coding sequence. The plasmid coding for GFP provided the basis for normalization and copy number calculation. We observed different numbers of GFP copy integration in the genomes of the obtained cell lines (Fig. S1A). However, cells that demonstrated bright fluorescence contained a decreased GFP copy number (Fig. S1A) in comparison to those with a low level of fluorescence (Fig. 1B), thus demonstrating that the significant difference in the GFP fluorescence was due to reasons independent of the variation in copy number of the particular cassette in the genome of the cell line.

We performed 5′-RACE analysis to confirm that transcription was driven solely by promoters from the expression cassette in PhTR, PSFFV (Fig. S1B) and PSFFV-PhTR_b and PSFFV-PhTR (Fig. S1C) cell lines. To detect the 5′-ends of the transcripts, they were reverse-transcribed from the primer complementary to the specific region within hTR (Table S1) followed by lengthening of the 5′-end by terminal transferase and PCR-amplification. As expected, in the cell lines expressing the bicistronic construct under control of the hTR- or SFFV promoter, only a single product (Fig. S1B) was detected that started at the +1 position of hTR. In the case of cells with the SFFV-hTR-coupled promoter, two forms of hTR that differed in terms of their 5′-ends (Fig. S1C) were detected. Sequencing of the obtained products demonstrated that the shorter form corresponded to the mature native 5′-end of hTR, and the longer one started from the SFFV promoter (the 5′-end contained the hTR promoter sequence). As mentioned above, the cells with the coupled promoter showed weak GFP fluorescence that increased in certain cell populations following a long cultivation. The 5′-RACE analysis showed that in cells with a weak GFP signal, transcription started predominantly from the hTR native promoter, but in the cells with an increased GFP signal, the SFFV promoter won the competition and directed more effective transcription (Fig. S1C). It should be mentioned that GFP fluorescence was reduced if transcription was terminated after the first gene. The shift in GFP fluorescence upon promoter switching suggested that the termination point was dependent on the promoter. Alternatively, mutations in the 3′-terminal region of hTR could prevent transcript cleavage after the first gene and thus promote the accumulation of bright GFP-positive cells. To answer this question, we sorted cells expressing the bicistronic construct under control of the coupled SFFV-hTR-promoter for the bright and nonbright fluorescent cell populations (Fig. 1C). The obtained cells were used for genomic DNA purification followed by amplification of the region corresponding to the hTR genomic region from the bicistronic construct and sequencing. No changes in sequences were found, thus indicating that the promoter choice determined the transcript cleavage during transcription termination.

**Figure 2:**
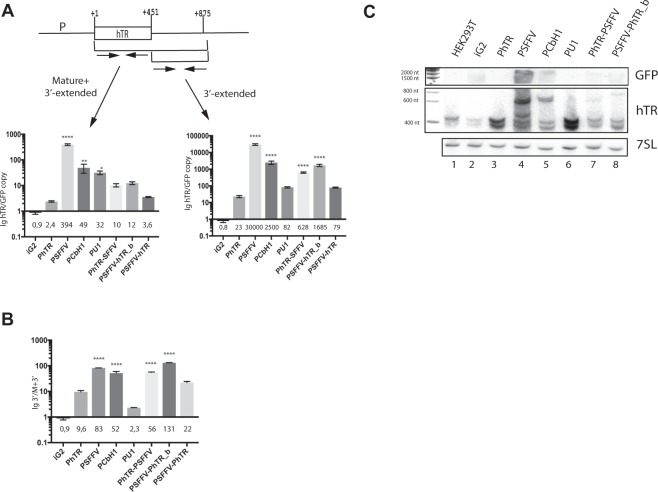
The promoter defines the termination of transcription of hTR. (**A**) Analysis of the expression of the total hTR (Mature + 3′-extended) (left panel) and its 3′-extended (3′-extended) (right panel) region was performed by RT-qPCR. The mean fold-change in mature or 3′-end-extended hTR was normalized to the reference gene (GAPDH mRNA) and compared with the hTR level in HEK293T cells. Additionally, we normalized the samples to the GFP copy number determined for the cells during transduction of the reporter construct. The mean values were calculated from triplicate RT-qPCR experiments of three biological replicates with bars representing the SE. The **** indicates P < 0,0001, **P < 0,01 and *P < 0,1 by Sidak’s multiple comparisons test. (**B**) The ratio of the level of the 3′-extended region of hTR to the total hTR level was calculated to compare the transcription termination and processing rate independently of the different expression levels directed by various promoters in reporter constructs. The **** indicates P < 0,0001 by Sidak’s multiple comparisons test. (**C**) Northern blot analysis of the reporter constructs. The top panel shows hybridization to GFP specific probes to demonstrate the bicistronic construct expression. The middle panel shows hybridization hTR-specific probes to demonstrate the variations in hTR forms expressed from different promoters. The bottom panel was used as a loading control (7SL RNA) to demonstrate equal amounts of total RNA.

To determine whether the transcription termination positions were dependent on the promoter, we quantified the transcript amounts of either hTR or the intergenic regions in cell lines carrying the constructs under the control of different promoters using RT-qPCR analysis. Primers specific to the mature form of hTR and to the 3′-end-extended form of hTR were used (Figs 2A and S2A, Table S2). The data were normalized to the GFP copy integrated in the genome for each cell line. Increased levels of hTR (independent of the 3′-end) by 2-, 5-, 394-, 49-, 32-, 10-, 12,5- and 3,6-times were observed for PhTR, PSFFV, PCbH1, PU1, PhTR_PSFFV, PSFFV-hTR_b and PSFFV-hTR, respectively, in comparison to the control iG2-cell line expressing empty vector (Fig. 2A, left histogram). The level of the 3′-extended form of hTR was increased by 23-, 30000-, 2500-, 82-, 628-, 1685- and 79-fold for PhTR, PSFFV, PCbH1, PU1, PhTR-PSFFV, PSFFV-hTR_b and PSFFV-hTR, respectively, in comparison to the control iG2 cell line expressing empty vector (Fig. 2A, right histogram). We normalized the level of 3′-extended hTR (3′) to the total level of hTR transcript (M + 3′) to estimate the level of processing independently of the expression rate changes. We found that the level of 3′-extended hTR increased significantly in the case of Mediator-regulated promoters (PSFFV, PCbH1 and coupled PSFFV-PhTR_b) (Fig. 2B).

To obtain independent evidence that transcription termination in the case of hTR was dependent on the type of promoter, we performed a Northern blot analysis of the total RNA purified from model cell lines. We detected hTR with oligonucleotide probes specific to the hTR region (Table S3). Northern blot revealed several additional bands corresponding to unprocessed hTR in the cells expressing hTR under the control of promoters regulated by the Mediator PSFFV-PhTR-, PSFFV- and PCbH1-promoters (Fig. 2C, middle panel). Correctly matured hTR was observed in all cell lines wherein expression was driven by Integrator-regulated promoters: PhTR and PU1 (Fig. 2C, middle panel). Indeed, only the mature form of hTR could be detected in the cell lines PhTR and PU1 (Fig. 2C, middle panel), but not GFP mRNA (second cistron, analyzed with the probe specific to GFP mRNA) (Fig. 2C, top panel). Different amounts of bicistronic mRNA were observed in all cell lines expressing hTR driven by the Mediator-dependent promoters (PSFFV, PCbH1 and coupled promoters) (Fig. 2C, top panel). Two additional products (approximately 500 and 600 nt) were detected when hTR was expressed under the control of the SFFV promoter (Fig. 2C, middle panel). CbH1, PhTR-PSFFV and PSFFV-PhTR promoter-dependent transcription led to the accumulation of only one additional product (approximately 600 nt) (Fig. 2C, middle panel). We re-probed the Northern blot with probes specific to 7SL RNA as a loading control to confirm that the same number of cells was used in these experiments (Fig. 2C, bottom panel). The Northern blot data demonstrated only the mature form of hTR in the PhTR and PU1 cell lines (Fig. 2C). However, when the hTR expression was controlled by Mediator-dependent promoters, the accumulation of hTR containing RNA products of different lengths could be detected by Northern blotting, which could appear as the result of an interference in the correct termination or subsequent processing of RNA products or both (Fig. 2C). A clear correlation between the fluorescence and hTR processing levels (estimated either by RT-qPCR or Northern blotting) could be seen (Figs 1C and 2B). The processing of hTR was significantly affected in the bright cells and only slightly in cells with a low fluorescent signal. These data confirmed that efficient termination of hTR only occurred when transcription was controlled by promoters regulated by Integrator, which allow us to conclude that transcription termination of hTR occurred in a promoter-dependent mode and that Integrator should play the main regulatory role in the correct transcription and processing of hTR.

### Expression of Integrator subunits is regulated by a feedback loop

To confirm that Integrator was indeed a key regulator of correct hTR processing, knockdowns of different Integrator subunits were applied to evaluate the correlation between the level of the Integrator subunit and the accuracy of hTR processing.

INTS1 is the largest component of Integrator, and it may play a role as a scaffold that coordinates the interaction of regulatory factors with RNA polymerase II during transcription^18^. INTS9 and INTS11 are involved in 3′-end processing of the synthesized transcripts. INTS11 is a homolog of CPSF73, the major cleavage factor for mRNA genes that participates in transcript cleavage, while INTS9 is a homolog of CPSF100 that is involved in coordination of the cleavage of nascent transcript-promoting transcription termination and regulation of the pause/release of RNA-polymerase II^18^.

**Figure 3:**
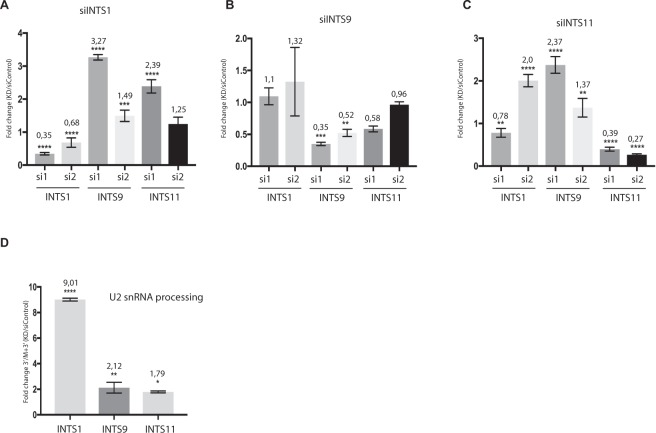
Expression of Integrator subunits is regulated by a feedback loop and affects 3′-end formation of U2 snRNA. (**A**–**C**) Total RNA prepared from HEK293T cells treated with siRNAs against the Integrator subunits INTS1 (**A**), INTS9 (**B**) and INTS11 (**C**) was subjected to RT-qPCR to assess the mRNA levels of INTS1, INTS9, INTS11 and GAPDH. The mean fold-change in the analyzed RNA was normalized to the control siRNA and to the reference gene. Mean values were calculated from triplicate RT-qPCR experiments of three biological replicates, with bars representing the SE. (**D**) The total RNA prepared from HEK293T cells treated with siRNAs against the Integrator subunits INTS1, INTS9, INTS11 was subjected to RT-qPCR to measure the levels of the total U2 snRNA transcript (M + 3′) and the 3′-end-extended form of U2 snRNA (3′). The ratio of the level of the 3′-extended region of U2 snRNA to the total U2 snRNA level was calculated to compare the transcription termination and processing rate. Mean values were calculated from triplicate RT-qPCR experiments of three biological replicates, with bars representing the SE. The **** indicates P < 0,0001, ***P < 0,001, **P < 0,01 and *P < 0,1 by Sidak’s multiple comparisons test.

Knockdowns of Integrator components were performed in wild-type HEK293T cells to avoid artificial changes in the expression levels of hTR from the model constructs in the created cell lines with the help of siRNAs that have been previously described to be efficient in the case of selected Integrator subunits^18–20^ (Table S4). siRNA specifically targeting Firefly luciferase mRNA was used as a nonspecific control. We confirmed the specificity of siRNA action using sets of two siRNAs for each gene and demonstrated the very similar effects of the gene expression inhibition (Fig. 3A–C). Interestingly, we found that depletion of particular Integrator subunits led to increases in the amounts of several others. Depletion of the INTS1 subunit using a specific siRNA (Fig. 3A) caused a simultaneous significant increase in the expression level of the other analyzed Integrator’s subunits, INTS9 and INTS11 (Fig. 3A). The same tendency was observed for the knockdown of INTS11 (Fig. 3C). The level of INTS1 and INTS9 increased when INTS11 expression was inhibited (Fig. 3C). However, the knockdown of INTS9 did not significantly influence the decrease in INTS1 and INTS11 (Fig. 3B). Similar effects were observed for each of the two siRNAs for the same Integrator subunit. Thus, some Integrator subunits may somehow influence the level of several others. This finding requires further investigation to reveal the mechanism underlying such possible regulation.

### Depletion of Integrator subunits affects 3′-end formation of hTR

Integrator is known as a major player in the 3′-end formation of snRNAs. To validate whether the knockdown of a particular Integrator subunit indeed disrupted snRNA processing under our experimental conditions, the levels of the mature and unprocessed 3′-extended regions of U2 snRNA were determined by RT-qPCR (Fig. S3A,B). Primers specific to the mature form were applied to allow estimation of the total U2 snRNA transcript level (mature form together with read-through termination site transcript). The level of only the read-through transcript was detected with primers specific to the 3′-end-extended region of U2 snRNA. We observed dramatic increases in both the level of mature U2 snRNA and the 3′-end-extended transcript (Fig. S3A,B), in agreement with previously published data^18^. To compare the amount of processed and mature RNA, we normalized the amount of 3′-end-extended transcript of the U2 snRNA (3′) to the amount of total U2 snRNA (M + 3′). The amount of unprocessed U2 snRNA increased 9-, 2.1- and 1.8-times after knockdown of INTS1, INTS9 and INTS11, respectively (Fig. 3D), in comparison to cells treated with the siRNA targeting Firefly luciferase mRNA, which was used as a nonspecific control.

**Figure 4:**
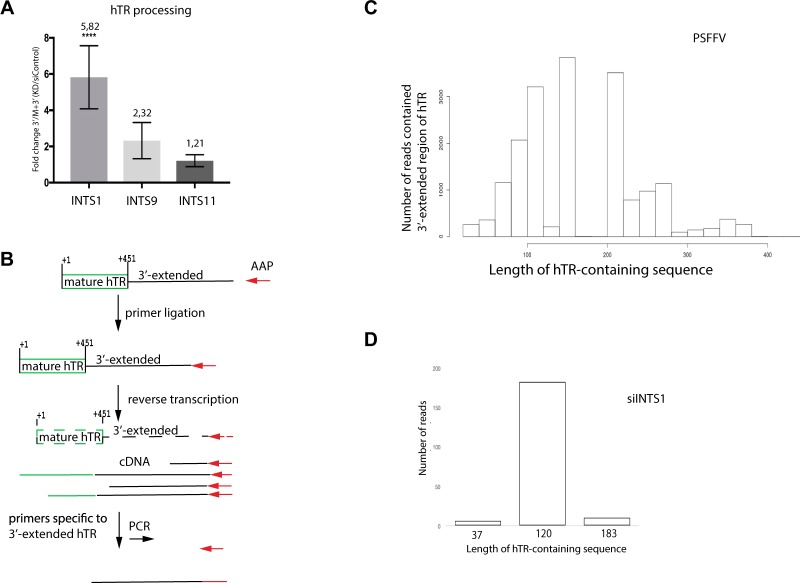
Integrator is involved in hTR transcription termination. (**A**) Total RNA prepared from HEK293T cells treated with siRNAs against the Integrator subunits INTS1, INTS9, and INTS11 was subjected to RT-qPCR to measure the levels of total hTR transcript (M + 3′) and the 3′-end-extended form of hTR (3′). The ratio of the level of the 3′-extended region of hTR to the total hTR level was calculated to compare the transcription termination and processing rate. Mean values were calculated from triplicate RT-qPCR experiments of three biological replicates, with bars representing the SE. The **** indicates P < 0,0001 by Sidak’s multiple comparisons test. (**B**) Schematic of the 3′-RACE approach used for 3′-end detection of the 3′-end-extended hTR transcript. (**C**,**D**) A histogram illustrating the distribution of the lengths of hTR-containing reads obtained for HEK293T cells expressing hTR under the control of the SFFV-promoter (**C**) and for HEK293T cells treated with siRNA targeting the INTS1 gene (**D**). The 3′-ends were determined using a RNA-ligase-mediated 3′-RACE approach, followed by pyrosequencing.

To quantify the effect of INTS1, INTS9 and INTS11 inhibition on hTR processing, we performed a similar RT-qPCR analysis of the level of total hTR (M + 3′) and the 3′-end-extended transcripts of hTR (3′) using the same total RNA from HEK293T cells treated with control siRNA and siRNA specific to Integrator subunits. Since the depletion of Integrator had an effect on the splicing transcription machinery of the cell, to compare the data on hTR processing obtained for different cell lines, the amount of 3′-end-extended hTR (3′) was normalized to the total amount of hTR gene transcripts (M + 3′). We observed an enrichment of the 3′-end-extended form of hTR by 5,8-, 2,3- and 1,2-times in cells treated with siRNAs targeting INTS1, INTS9 and INTS11, respectively, in comparison to those treated with siRNA targeting Firefly luciferase mRNA (Fig. 4A).

The 3′-end boundary sequence of the hTR primary transcript could only be identified under conditions when the extended transcript could be detected in the cell. We decided to use cells in which hTR processing was clearly affected: cells expressing the bicistronic construct under control of the hTR and SFFV promoter and wild-type cells treated with siRNA targeting INTS1, since INTS1 is a platform subunit that keeps together the Integrator complex and may influence both transcription termination and subsequent 3′-end RNA processing. The 3′-RACE approach followed by pyrosequencing was used to determine the 3′-end of the hTR containing RNA fragments from the above-mentioned cell lines. As a first step in this procedure, we performed adapter primer ligation to the total RNA, followed by reverse transcription with a primer complementary to the ligated adaptor (Fig. 4B). The obtained products were amplified with a forward primer (Fig. 4B) specific to the region located immediately downstream of the mature form of hTR (primer 3′-RACE Fw, Table S1) in an attempt to identify long precursors of hTR and to avoid amplification of the mature hTR and its previously described oligo(A)-precursor^13,14,21^. We observed a wide distribution of hTR-containing products with a lengthened 3′-end from 30 up to 400 nucleotides when we expressed hTR under the control of the SFFV promoter (Fig. 4C, Table S5) and no sequences for the 3′-end-lengthened hTR in cells carrying the bicistronic construct under control of the hTR promoter (Table S5). These data confirmed the appearance of the product observed by Northern blot analysis (Fig. 2C) and demonstrated that the native hTR promoter led to accurate transcription termination and maturation of hTR despite the context of the bicistronic construct. Conversely, the SFFV promoter was unable to accomplish the correct maturation of hTR.

To define the position of premature termination upon Integrator depletion, the 3′-RACE procedure followed by pyrosequencing was performed for wild-type cells treated with siRNA targeting the INTS1 subunit of Integrator. Knockdown of INTS1 resulted in an accumulation of 3′-end-extended hTR for 120 nucleotides (the full length of the particular hTR transcript was 571 nucleotides) in HEK293T cells (Fig. 4D, Table S5), and no reads containing the extended hTR sequence were obtained after treatment with siRNA specific for the Firefly luciferase coding sequence (this siRNA served as a negative control) (Table S4). These data support our previous findings that Integrator is responsible for the coordination of processing events after transcriptional termination of hTR.

Taken together, our results validate that Integrator is involved in the termination of hTR gene transcription and may act as a coordinator of transcription and processing events during hTR biogenesis.

## Discussion

In this study, we uncovered an unexpected role for the Integrator complex in the promoter-dependent maturation of hTR. Here we have shown that knockdown of Integrator results in the accumulation of long hTR transcripts and determines the length of this hTR transcript.

Telomerase RNAs from various organisms demonstrate a high level of diversity in their sequences and structures. Moreover, the mechanisms of telomerase RNA maturation differ considerably. The 3′-spliceosomal cleavage of primary transcripts in diverse yeast and filamentous fungi results in correctly processed telomerase RNA^22^. Alternatively, investigations of *S*. *cerevisiae* telomerase RNA (TLC1) transcription termination and processing have revealed that the NNS-complex containing Nrd1, Nab3 and Sen1 facilitates transcript cleavage^23^ and should recruit the exosome complex involved in processing or degradation^24^. Nrd1 and Nab3 also interact with the TRAMP complex that facilitates the oligoadenylation of the TLC1 transcript required for correct processing^24,25^. It is known that the NNS-complex facilitates 3′-end formation of sn- and snoRNAs in *S*. *cerevisiae*^25,26^. Additionally, hTR shares structural properties with H/ACA-box snoRNAs^11^, but the gene organization of hTR is very similar to the gene organization of the *S*. *cerevisiae* TLC1 gene. The primary transcript of hTR is processed by exosome trimming^13^. Similarities in the gene organization and mechanism of processing of the primary transcript have allowed us to propose a common mechanism for the termination of transcription. Although human cells contain a homolog of Sen1, Senataxin, structural homologs of Nab3 and Nrd1 have not been identified in vertebrates, and their role in the 3′-end formation of snRNAs has been attributed to the Integrator complex^20^. The Integrator complex plays an important role in the regulation of transcription initiation and the 3′-end processing of many classes of RNAs, such as snRNAs, non-polyadenylated mRNAs (histone mRNAs) and the mRNAs of the Integrator target gene^19^. INTS1 has been proposed to facilitate the scaffolding for the interaction of the Integrator complex^27^ and other factors with RNA polymerase II, while INTS9 and INTS11 facilitate 3′-end formation of snRNAs and some mRNAs^20^. Integrator regulates transcription in a promoter-dependent manner^19^.

In the present study, expression of hTR under the control of the non-cognate promoter resulted in the appearance of the 3′-end-extended primary transcript with the help of model expression constructs (Fig. 2B,C), in agreement with previously published data^12^, supporting the phenomenon of promoter-dependent transcription termination. Integrator coordinates different regulatory factors at the synthesized transcript and interacts with other complexes that participate in the regulation of transcript synthesis and processing^28^. CBC-containing complexes associate with the m7G-cap structure at the 5′-end of newly synthesized transcripts and, in turn, recruit specific factors that either determine the appearance of the correct 3′-end or RNA degradation^29^. The very high rate of exchange for proteins associated with CBC plays an important role in decision-making concerning RNA fate. ZC3H18 binding to the CBCA complex attracts the NEXT exosome complex to the transcript, which results in RNA decay^30^. Integrator binding to the CBCA complex through an interaction with NELF facilitates effective 3′-end formation of snRNAs^31^.

**Figure 5:**
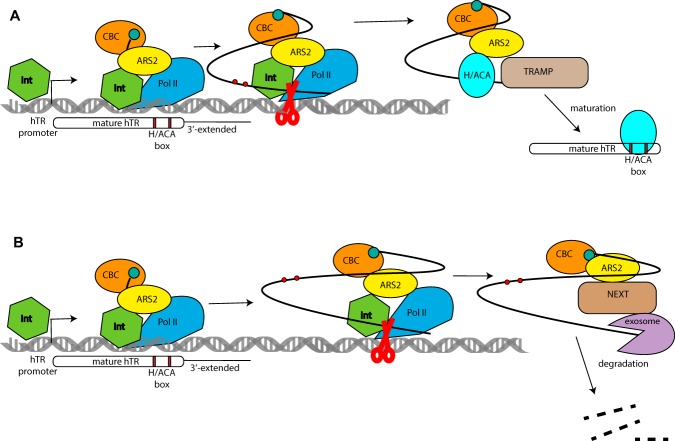
Schematic illustrating the regulation of hTR biogenesis by Integrator.

The accumulation of 3′-end-extended forms of hTR with a similar size upon knockdown of CBCA, NEXT^13^ and Integrator components (Fig. 4D) allowed us to propose that decisions regarding the fate of hTR should be made co-transcriptionally. We suppose that effective transcription termination facilitated by Integrator results in recruitment of the TRAMP-complex and correct maturation of hTR (Fig. 5A). However, in cases in which RNA polymerase II read through the termination signal, the 3′-end-extended transcript appeared, thus weakening the interaction of Integrator with CBCA and leading to the recruitment of NEXT to the 3′-extended hTR to degrade it (Fig. 5B).

Cells regulate the physiological level of hTR very carefully. Competition between degradation and processing adjusts the amounts of correctly matured hTR. We have shown that the Integrator complex is involved in the regulation of hTR transcription termination and may discriminate the primary transcript that should be processed into mature hTR from aberrant transcripts that should be degraded.

## Materials and Methods

### Cell culture and expression constructs

To create the PSFFV-PhTR and PhTR constructs, the hTR genomic region was produced by PCR with the oligonucleotides PSFFV-PhTR Fw 5′-GGACGCATCCCACTGAGCC-3′ or PhTR Fw 5′- GAATTCGGGTTGCGGAGGG-3′ and Rv 5′-GGGCCCGGAACACACTTTCCAATG-3′. The obtained products were digested with *Eco*RI and *Bsp*120I and cloned into the LeGo-iG2 vector^32^. To produce the PhTR construct, the PSFFV-PhTR construct was digested by *Eco*RI and *Nhe*I, processed by T4 DNA-polymerase and ligated. To clone the PCbH1 construct, the CbH1-promoter was obtained by restriction of the pX458^33^ vector. pX458 was digested with *Age*I, processed with T4 DNA polymerase and digested with *Xba*I. The obtained product was ligated with PSFFV construct treated with *Eco*RI, processed with T4 DNA polymerase and digested with *Nhe*I. To produce the PU1 construct, pBS-U1-hTR^34^ plasmid was digested with *Spe*I, followed by processing with T4 DNA-polymerase and digestion by *Xba*I. The obtained product was ligated to the PhTR construct treated with *Eco*RI, T4 DNA polymerase and *Xba*I. The obtained constructs were verified by sequencing. Human HEK293T cells were cultured in DMEM supplemented with 10% fetal bovine serum and transiently transfected with the vectors and lentiviral plasmids using the calcium phosphate method. The lentiviral particles were harvested and used for HEK293T cell infection according to a previously published protocol^32^. The cells were analyzed using the EVOS FL Imaging System (Thermo Fisher Scientific) and sorted using a FACSAriaIII cell sorter (Beckton Dickinson). The cells were tested for mycoplasma contamination and found to be negative.

### RNAi analysis

The siRNA validation and working concentration were determined after 48 hours of cell treatment. For siRNA knockdown, the cells were treated three times (2-day interval) with siRNA using Lipofectamine RNAiMAX (Invitrogen) according to the manufacturer’s instructions. Primary treatment was performed as reverse transfection, and the second and third treatment were performed as forward transfection.

### RT-qPCR analysis

Total RNA was prepared using the PureLink RNA Mini Kit (Ambion) according to the manufacturer’s instructions, followed by treatment with DNase I (Thermo Scientific) at 37 °C for 30 min. The RNA was reverse-transcribed to cDNA using the Maxima First Strand cDNA Synthesis Kit for RT-qPCR (Thermo Scientific) with random primers at 60 °C for 1 hour. All real-time PCR reactions were performed using the primers described in Table S2. The PCR efficiency was determined in reactions with plasmids for each primer pair specific to hTR or with genomic DNA using primer pairs specific to U2 snRNA (Fig. S2A,C). To control DNA contamination, total RNA that was not treated with reverse transcriptase was used as a template in the PCR reaction (Fig. S2D). Then, qPCR was performed using a CFX96 Real-Time system (Bio-Rad) with Maxima SYBR Green/ROX qPCR Master Mix (2X) (Thermo Scientific). The PCR cycle parameters were as follows: 95 °C for 2 min; 40 cycles of denaturation at 95 °C for 30 sec, annealing at 60 °C for 30 sec, and an extension step at 72 °C for 60 sec. All PCR amplifications were performed in triplicate and repeated in three independent experiments. All data were normalized to multiple reference genes, GAPDH mRNA, B2M mRNA and U2 snRNA^35^, in accordance to the “Guide to Performing Relative Quantitation of Gene Expression Using Real-Time Quantitative PCR” by Applied Biosystems. To calculate the rate of processing (3′/M + 3′) under conditions in which the Integrator subunits were knocked down, the levels of the 3′-end-extended substantial transcript (3′) were normalized to the total substantial transcript amount (M + 3′).

### Northern blot analysis

RNA samples were mixed with an equal volume of 2× formamide loading buffer (93% formamide, 0.1× Tris/Borate/EDTA (TBE), 30 mM EDTA, 0.03% bromophenol blue, and 0.03% xylene cyanol), heated at 95 °C for 5 min and then electrophoresed on a 4% polyacrylamide/7 M urea/1× TBE denaturing gel. Then, the RNA was transferred onto a Hybond^TM^-N^+^ membrane (GE Healthcare) in 1× BE at 1 A for 1–2 h and cross-linked to the membrane under UV light at 254 nm and 1200 × 100 μJ/cm^2^. The membrane was pre-hybridized in Church buffer (0.5 M Na_2_HPO_4_-H_3_PO_4_ buffer pH 7.2, 1 mM EDTA, 7% SDS, and 1% BSA) at 35 °C for 30 min and then in Church buffer with 5′-end-labeled oligo probes^36^ (Table S3) at 35 °C overnight. Subsequently, the membrane was washed once with 2× SSC and 0.1% SDS at 50 °C for 20 min and then twice with 0.1× SSC and 0.1% SDS at 50 °C for 20 min each time. The signals on the membrane were detected with a Typhoon FLA 9500 (GE Healthcare). Probes specific to GFP mRNA^37^ were labeled with terminal deoxynucleotidyl transferase (Thermo Scientific).

### RNA ligase-mediated 3′-RACE with pyrosequencing

Library preparation was performed according to a protocol similar to one previously described^21^. The oligonucleotides used for library preparation are listed in Table S1. The ligation reactions contained 2 μg of DNase I that had been treated and purified using the PureLink RNA Mini Kit (Ambion) for total RNA and 100 pmol of the RNA-adapter primer. The ligation reaction was incubated at 16 °C for 18 hrs and purified using the PureLink RNA Mini Kit (Ambion). RNA ligation reactions were annealed with 1 mmol RT-primer (3′RACE cDNA) and 0.5 mM dNTPs at 65 °C for 5 min, cooled down to room temperature and transferred to 60 °C for reverse transcription in a 20-ml volume containing 1x RT Buffer, 20 U of RiboLock RNase Inhibitor (Thermo Scientific), and 200 U of Maxima Reverse Transcriptase (Thermo Scientific) for 60 min. The first round of PCR was performed with 0.6 mM 3′-RACE Fw and 3′-RACE Rv in a 20-μl volume containing 10 μl of PCR Master Mix (2X) (Thermo Scientific) using the following program: 95 °C for 5 min; 35 cycles of 95 °C for 30 s, 63 °C for 30 s, and 72 °C for 1 min and 20 s, and a final extension at 72 °C for 10 min. Then, 2 ml of the first-round PCR product was used as template for the second-round PCR reaction containing 0.6 mM of the respective indexed primers as indicated in Table S1 and 25 μl of the PCR Master Mix (2X) (Thermo Scientific). The following program was used: 95 °C for 5 min; 35 cycles of 95 °C for 30 s, 63 °C for 30 s, and 72 °C for 1 min and 20 s; and a final extension at 72 °C for 10 min. Second-round PCR products were loaded onto a 1.5%-agarose gel, and amplicons of 100–400 bps were excised for purification (Zymoclean Gel DNA Recovery Kit).

The PCR fragments were sequenced with a Roche Genome Sequencer (GS FLX) using the Titanium XL + protocol according to the manufacturer’s instructions. Reads with mismatches to the primer sequences as well as those containing ambiguous nucleotides were excluded from the analysis. Reads containing hTR sequences were identified using a BLASTN search.

### 5′-RACE analysis

The 5′-RACE analysis was performed using the 5′RACE System for Rapid Amplification of cDNA Ends (Invitrogen) according to the manufacturer’s recommendations. The oligonucleotides used for the 5′-RACE analysis are listed in Table S1. DNase I-treated total RNA was reverse-transcribed into cDNA using the Maxima First Strand cDNA Synthesis Kit for RT-qPCR (Thermo Scientific) at 60 °C for 1 hour. The obtained cDNA was lengthened by terminal deoxynucleotidyl transferase (Thermo Scientific), followed by PCR amplification with PCR Master Mix (2X) (Thermo Scientific). The following program was used: 95 °C for 5 min; 35 cycles of 95 °C for 30 s, 62 °C for 30 s, and 72 °C for 1 min; and a final extension at 72 °C for 10 min. The obtained amplicons were loaded on a 1.5%-agarose gel, and the products were excised for purification (Zymoclean Gel DNA Recovery Kit), which was followed by sequencing.

## Supplementary information

Supplementary info_Rubtsova
