# Supplementary material for: Integrator is a key component of human telomerase RNA biogenesis
Source: Sci Rep. 2019 Feb 8;9:1701. doi: 10.1038/s41598-018-38297-6 (PMC6368637; doi:10.1038/s41598-018-38297-6)
Supplement: Supplementary file 1 — Supplementary info_Rubtsova [file 41598_2018_38297_MOESM1_ESM.pdf]

Integrator is a key component of human telomerase RNA biogenesis

M. P. Rubtsova[1,2,#], D. P. Vasilkova[2,#], M.A.Moshareva[2], A. N. Malyavko[1,2], M. B. Meerson[2], T. S. Zatsepin[1,2], Y. V. Naraykina[1,2], A.V. Beletsky[3], N. V. Ravin[3], and O. A. Dontsova[1,2,4]

Supplementary information

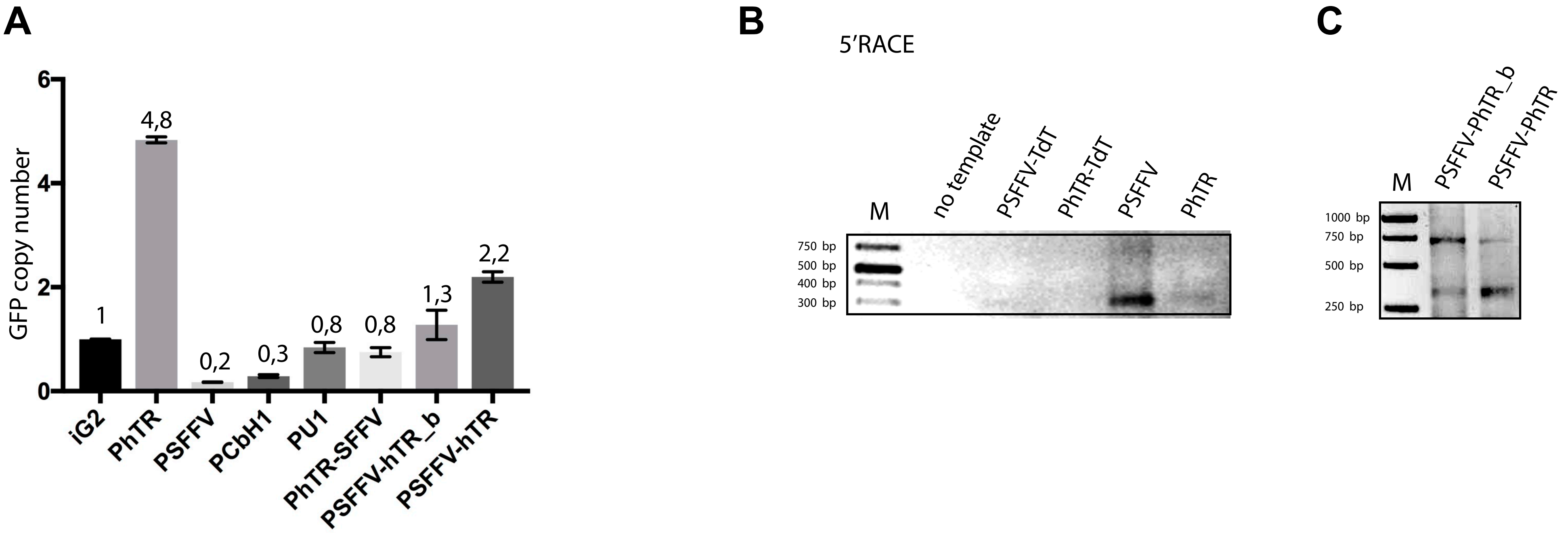
A
GFP copy number
6
4
2
0
4,8
1
0,2
0,3
0,8
0,8
1,3
2,2
iG2
PhTR
PSFFV
PCbH1
PU1
PhTR-SFFV
PSFFV-hTR_b
PSFFV-hTR
B
5’RACE
M
no template
PSFFV-TdT
PhTR-TdT
PSFFV
PhTR
750 bp
500 bp
400 bp
300 bp
C
M
PSFFV-PhTR_b
PSFFV-PhTR
1000 bp
750 bp
500 bp
250 bp

Fig. S1

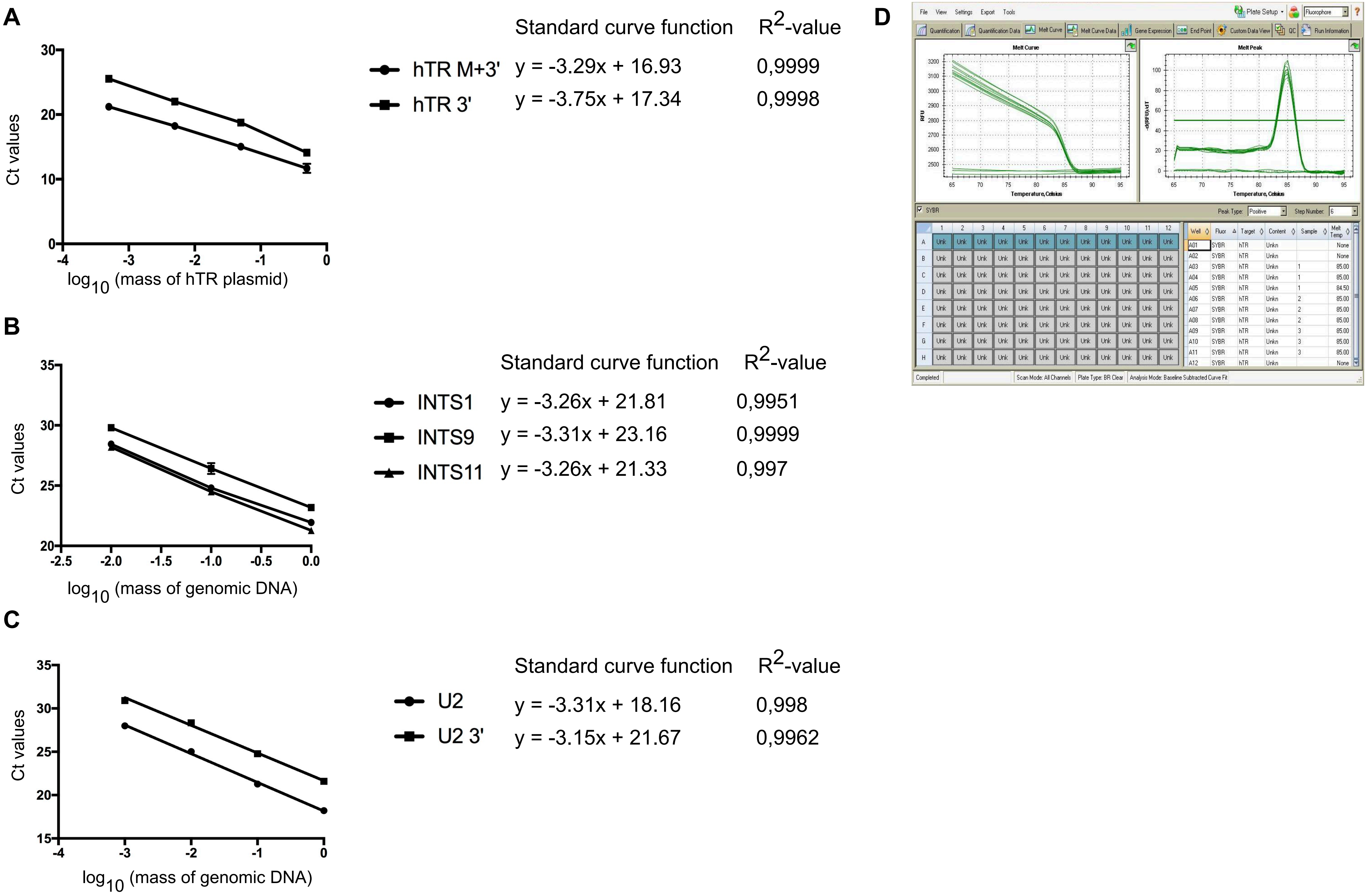
A
Ct values
log10 (mass of hTR plasmid)
Standard curve function
R2-value
hTR M+3'
y = -3.29x + 16.93
0,9999
hTR 3'
y = -3.75x + 17.34
0,9998
B
Ct values
log10 (mass of genomic DNA)
Standard curve function
R2-value
INTS1
y = -3.26x + 21.81
0,9951
INTS9
y = -3.31x + 23.16
0,9999
INTS11
y = -3.26x + 21.33
0,997
C
Ct values
log10 (mass of genomic DNA)
Standard curve function
R2-value
U2
y = -3.31x + 18.16
0,998
U2 3'
y = -3.15x + 21.67
0,9962
D
Melt Curve
Melt Peak
Temperature, Celsius

Fig. S2

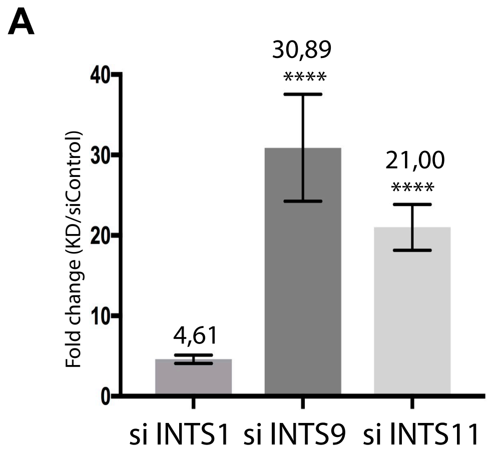
A
30,89
****
21,00
****
4,61
40
30
20
10
0
Fold change (KD/siControl)
si INTS1
si INTS9
si INTS11

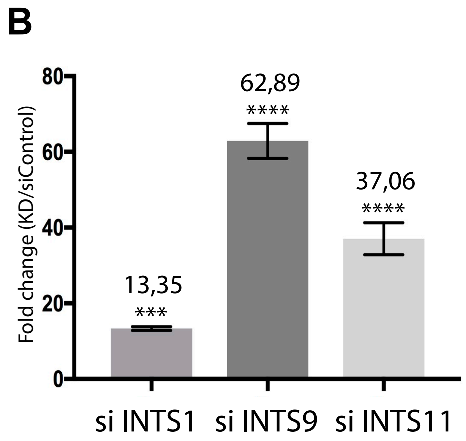
B
62,89
****
37,06
****
13,35
***
80
60
40
20
0
Fold change (KD/siControl)
si INTS1
si INTS9
si INTS11

Fig. S3

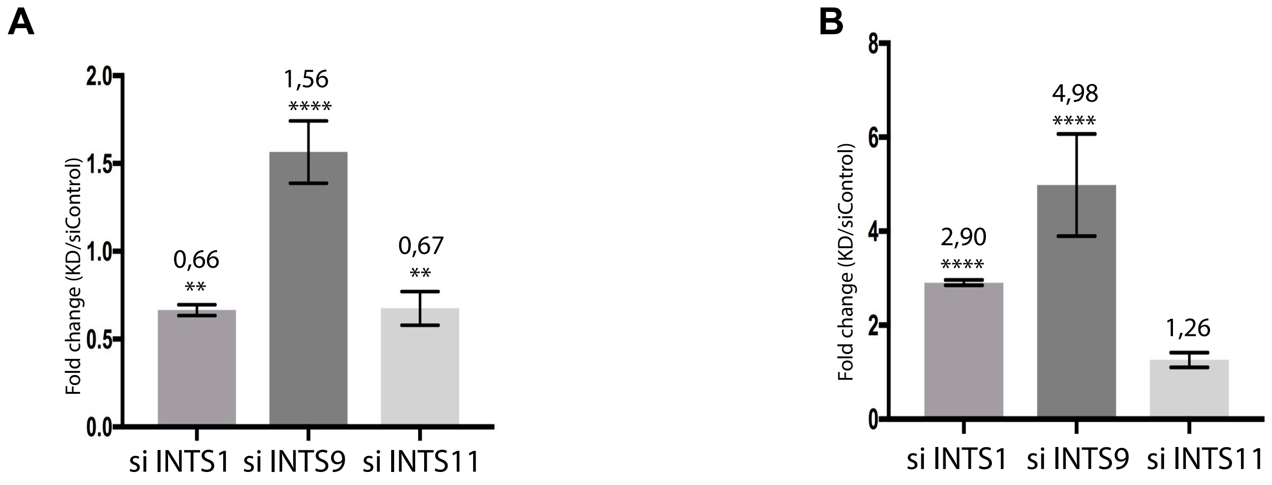
A
Fold change (KD/siControl)
2.0
1.5
1.0
0.5
0.0
0,66
**
1,56
****
0,67
**
si INTS1
si INTS9
si INTS11
B
Fold change (KD/siControl)
8
6
4
2
0
2,90
****
4,98
****
1,26
si INTS1
si INTS9
si INTS11

Fig. S4

Figure S1. Transcription termination of human telomerase RNA depends on the promoter type. (A) Genomic DNA was subjected to RT-qPCR analysis to calculate the GFP copy number integrated in the genomes of reporter cell lines. (B,C) Total RNA from cells expressing hTR under the control of hTR- (PhTR) (B), SFFV- (PSFFV) (B) and coupled SFFV-hTR-promoters (PSFFV-PhTR_b, PSFFV-PhTR) (C) was reverse-transcribed with the primer 5'-RACE cDNA (Supplementary Table 1), followed by lengthening using terminal deoxynucleotidyl transferase. The obtained cDNA was amplified, and the amplicons were analyzed using an agarose gel. As a control, cDNA untreated with terminal deoxynucleotide transferase was used for PCR amplification (-TdT lanes) (B).

Figure S2. Validation of the primers used for RT-qPCR analysis. (A-C) Examination of the efficiencies of the qPCR primer pairs. Each standard curve was generated by plotting the Ct values of the PCR reactions against the logarithm of the mass of genomic DNA for *U2*, *GAPDH*, *INTS1, INTS9*, *INTS11*, or the plasmid for hTR added to base 10. The slopes of the standard curves were all close to -3.3, which is the theoretical value for a perfectly efficient primer pair. The function and R^2 values for each curve are shown. (D) An example of the analysis of the qPCR primer pair specificities. Amplification curves of the qPCR with and without template are shown. The PCR dissociation (melt) curves are shown. Reverse transcription followed by PCR analysis was performed with samples A3-A11, and PCR analysis without reverse transcription was performed with samples A1, A2, A12.

Figure S3. Expression of Integrator subunits affects 3'-end formation of U2 snRNA. (A,B) Total RNA prepared from HEK293T cells treated with siRNAs against the Integrator subunits INTS1, INTS9 and INTS11 was subjected to RT-qPCR to measure the levels of total U2 snRNA transcript (M+3') (A) and the 3'-end-extended form of U2 snRNA (3') (B). The mean fold-change in the analyzed mRNA was normalized to the control siRNA for the total U2 snRNA transcript (M+3') and the 3'-end-extended form of U2 snRNA (3'). Mean values were calculated from triplicate RT-qPCR experiments of three biological replicates, with bars representing the SE. The **** indicates P<0,0001 by Sidak's multiple comparisons test.

Figure S4. Integrator is involved in hTR transcription termination. (A,B) The total RNA prepared from HEK293T cells treated with siRNAs against the Integrator subunits INTS1, INTS9 and INTS11 was subjected to RT-qPCR to measure the levels of total hTR transcript (M+3') (A) and the 3'-end-extended form of hTR (3') (B). The mean fold-change in the analyzed mRNA was normalized to the control siRNA for the total hTR transcript (M+3') and the 3'-end-extended form of hTR (3'). Mean values were calculated from triplicate RT-qPCR experiments of three biological replicates, with bars representing the SE. The **** indicates P<0,0001 and *** -- P<0,001 by Sidak's multiple comparisons test.

Table S1. Oligonucleotides used for 5'-RACE and RNA ligase mediated 3'-RACE with pyrosequencing, related to 5'-RACE and RNA ligase mediated 3'-RACE with pyrosequencing in Experimental procedures.

Name	Sequences	Note
5'RACE cDNA	CCCACCAACAGGAAAGCGAAC	RT-primer
AAP	GGCCACGCGTCGACTAGTACGGGIIGGGIIGGGIIG	PCR amplification of 5'-RACE
5'RACE Rv	GGCTGACAGAGCCCAACTCTT	PCR amplification 5'-RACE
RNA-adapter	pUCGUAUGCCGUCUUCUGCUUGidT	3'-linker for 3'-RACE
3'RACE cDNA	CAAGCAGAAGACGGCATACGA	RT-primer
3'RACE Fw	AGTTCGCTTTCCTGTTGGTG	First round PCR of 3'-RACE
3'RACE Rv	CAAGCAGAAGACGGCATACGA	
MID1 Fw	ACGAGTGCGTAGTTCGCTTTCCTGTTGGTG	Second round PCR of 3'-RACE
MID1 Rv	ACGAGTGCGTCAAGCAGAAGACGGCATACGA	
MID3 Fw	AGACGCACTCAGTTCGCTTTCCTGTTGGTG	
MID3 Rv	AGACGCACTCCAAGCAGAAGACGGCATACGA	
MID5 Fw	ATCAGACACGAGTTCGCTTTCCTGTTGGTG	
MID5 Rv	ATCAGACACGCAAGCAGAAGACGGCATACGA	
MID7 Fw	CGTGTCTCTAAGTTCGCTTTCCTGTTGGTG	
MID7 Rv	CGTGTCTCTACAAGCAGAAGACGGCATACGA	
MID9 Fw	TAGTATCAGCAGTTCGCTTTCCTGTTGGTG	
MID9 Rv	TAGTATCAGCACAAGCAGAAGACGGCATACGA	
MID14 Fw	CGAGAGATACAGTTCGCTTTCCTGTTGGTG	
MID14 Rv	CGAGAGATACCAAGCAGAAGACGGCATACGA	

Table S2. Oligonucleotides used in this study for reverse transcription and polymerase chain reaction, related to RT-qPCR in Experimental procedures

Name	Sequences	Note
M+3' Fw	GTGGTGGCCATTTTTTGTCTAAC	Total hTR
M+3' Rv	TGCTCTAGAATGAACGGTGGAA	
hTR 3' Fw	AGTTCGCTTTCCTGTTGGTG	3'-extended hTR (452-539)
hTR 3' Rv	AGGTTTGGGGGTTCACAAG	
U2 Fw	CGCTTCTCGGCCTTTTGGC	Total U2 snRNA
U2 Rv	GTGCACCGTTCCTGGAGGT	
U2 3' Fw	AGGGAGGTGAGAGACGGTAG	3'-extended U2 snRNA
U2 3' Rv	GTGGAAACGAAAGCGTGCC	
GAPDH Fw	TGCACCACCAACTGCTTAGC	Total *GAPDH* mRNA (Vandesompele et al., 2002)
GAPDH Rv	GGCATGGACTGTGGTCATGAG	
B2M Fw	TGCTGTCTCCATGTTTGATGTATCT	Total *B2M* mRNA (Vandesompele et al., 2002)
B2M Rv	TCTCTGCTCCCCACCTCTAAGT	
INTS1 Fw	GGACAAGAATTACATGGCCC	Total *INTS1* mRNA
INTS1 Rv	CTTTGGTTTGGGTGCCTCTG	
INTS9 Fw	CTCCTTGCTTACCACACACC	Total *INTS9* mRNA
INTS9 Rv	TGCAGAACTCTCCCACCATTC	
INTS11 Fw	CCTACTTCAGCGAGATGGTGG	Total *INTS11* mRNA
INTS11 Rv	TCTGGGAGGTGAAGAAGTTGG	

Table S3. Oligonucleotides used as probes in Northern blotting analysis, related to Northern blot in Experimental procedures.

Name	Sequences	Target	Note
N-hTR 1	GACTCGCTCCGTTCCTCTTC	hTR	(Xi and Cech, 2014)
N-hTR 2	GCTCTAGAATGAACGGTGGAA	hTR	
N-hTR 3	CCTGAAAGGCCTGAACCTC	hTR	
N-hTR 4	CGCCTACGCCCTTCTCAGT	hTR	
N-hTR 5	ATGTGTGAGCCGAGTCCTG	hTR	
GFP	ATAGACGTTGTGGCTGTTGTAGTTGTACTCCAGCTTGTGC	GFP	(Soboleski et al., 2005)
7SL	GGAGGTCACCATATTGATGCCGAACTTAGT	7SL	(Tseng et al., 2015)

Table S4. siRNAs used in this study.

Target	Sequences	Note
INTS1_1	AAUGAGGAUCCUGCAUAUGGAdTsdT	(Baillat et al., 2005)
INTS1_2	AAGAAACGACCUGAACUGUCAdTsdT	(Baillat et al., 2005)
INTS9_1	GAAAGCGGGUGAGCGAUGAdTsdT	(Skaar et al., 2015)
INTS9_2	GUGAACUCUGCCCUUAGUAdTsdT	(Jodoin et al., 2013)
INTS11_1	GCAAGAUCGCCGUAGACAAGAdTsdT	(Gardini et al., 2014)
INTS11_2	GCAGCCAUGUUCCAGAUUAAAdTsdT	(Gardini et al., 2014)

Table S5. Distrubution of the length of 3’-extended hTR transcript.

Name	Reads number	hTR reads number
HEK293T PhTR	2265	0
HEK293T PSFFV	61050	178
HEK293T siINTS1	4680	199
HEK293T siLuc	10560	0
